# The Electronic Structure and Secondary Pyroelectric Properties of Lithium Tetraborate

**DOI:** 10.3390/ma3094550

**Published:** 2010-09-01

**Authors:** Volodymyr.T. Adamiv, Yaroslav.V. Burak, David. J. Wooten, John McClory, James Petrosky, Ihor Ketsman, Jie Xiao, Yaroslav B. Losovyj, Peter A. Dowben

**Affiliations:** 1 Institute of Physical Optics, 23 Dragomanov Street, Lviv 79005, Ukraine; E-Mails: adamiv@ifo.lviv.ua (V.T.A.); burak@ifo.lviv.ua (Y.V.B.); 2 Air Force Institute of Technology, 2950 Hobson Way, Wright Patterson Air Force Base, OH 45433-7765, USA; E-Mails: tdjwooten@gmail.com (D.J.W.); John.Mcclory@afit.edu (J.M.); James.Petrosky@afit.edu (J.P.); 3 Deptment of Physics and Astronomy and the Nebraska Center for Materials and Nanoscience, Theodore Jorgensen Hall, 855 North 16th Street, University of Nebraska-Lincoln, Lincoln, NE 68588-0299 , USA; E-Mails: iketsman@unlserve.unl.edu (I.K.); Jie.Xiao@chemie.uni-erlangen.de (J.X.); 4 J. Bennett Johnston Sr. Center for Advanced Microstructures and Devices, Louisiana State University, 6980 Jefferson Highway, Baton Rouge, LA 70806, USA; E-Mail: ylosovyj@lsu.edu (Y.B.L.)

**Keywords:** oxide pyroelectric, band structure of insulators, surface states, secondary pyroelectric effects, lithium tetraborate

## Abstract

We review the pyroelectric properties and electronic structure of Li_2_B_4_O_7_(110) and Li_2_B_4_O_7_(100) surfaces. There is evidence for a pyroelectric current along the [110] direction of stoichiometric Li_2_B_4_O_7_ so that the pyroelectric coefficient is nonzero but roughly 10^3^ smaller than along the [001] direction of spontaneous polarization. Abrupt decreases in the pyroelectric coefficient along the [110] direction can be correlated with anomalies in the elastic stiffness $C_{33}^{D}$ contributing to the concept that the pyroelectric coefficient is not simply a vector but has qualities of a tensor, as expected. The time dependent surface photovoltaic charging suggests that surface charging is dependent on crystal orientation and doping, as well as temperature.

## 1. Introduction

The study of pyroelectricity has a long and rich history [1], but there are good reasons to suspect that the general models of pyroelectricity tend to be simplistic. Pyroelectricity is usually measured as a current that occurs with changing temperature along the direction of spontaneous polarization [1,2,3,4]. It is also important to note that all pyroelectric materials are piezoelectric, because the necessary spontaneous polarization only occurs in materials with a unique polar axis [2]. For a piezoelectric material we expect the charge density D_i_ to be related to the stress *X*_jk_ by [2]:

D_i_ = *d*_ijk_*X*_jk_ (1a)

where the piezoelectic coefficients, *d*_ijk_, form a third rank tensor. We should be able to alter the surface charge density, D_i_, by applying an electric field, as the strain is related to the applied electric field **E** by:

*x*_ij_ = *d*_ijk_E_k_ (2a)

with the stress related to strain, as described by Hooke’s law. The pyroelectric effect should, in fact, be a tensor because the surface charge density D_i_, induced by a change in temperature T, is also related to the change in static polarization *P*_S,i_, which in turn is related to the pyroelectric coefficient *p*_i_:

(3) $$D_{i}=\text{∆}P_{S,i}=\frac{dP_{S,i}}{dT}\text{∆}T=p_{i}\text{∆}T$$

From equations (1) and (2), with changes in temperature, there is an expected anisotropy of the electric constants and the resulting “stress” with temperature, particularly in a noncubic pyroelectric crystal. This implied tensor character makes equation (3), relating the surface charge density, D_i_, to the pyroelectric coefficients *p*_i_, over-simplistic. Although the pyroelectric coefficient is generally treated as a vector [2,3], there is implied tensor character present. This tensor character, higher than a first order, is due to the coupling to the stress-strain tensor and the accompanying tensor character of the piezoelectric effect. Indeed, it is recognized that there is a secondary pyroelectric effect that can occur if the pyroelectric crystal is allowed to deform along directions other than the polar direction [2], as has now been conclusively demonstrated [5] for lithium tetraborate (Li_2_B_4_O_7_). This is consistent with prior optical studies, which have provided some indications of an off-axis pyroelectric effect along crystal directions orthogonal to the polar axis of some translucent pyroelectric crystals [6].

This demonstration [5] of off-axis (secondary) pyroelectricity is expected and can be studied not only by conventional thermocurrent measurements, but by changes in the surface charging seen in photoemission. These latter measurements are based on detailed experimental characterization of the electronic structure in photoemission and inverse photoemission. More importantly, the identification of a surface electronic structure distinct from the bulk can be exploited to provide indications of surface piezoelectric effects that may differ from the bulk.

The value in characterizing the electronic band structure is not simply to assist in providing more signatures of pyroelectricity and piezoelectricity, but also help in assessing carrier mobilities. Lithium tetraborate remains one of the few piezoelectric materials for which there is a complete experimental band structure mapping [7]. This latter body of experimental data indicates that theory, in particular density functional theory, is not a completely infallible guide to the details of band structure of piezo-electric materials.

## 2. The Electronic Structure of Lithium Tetraborate

The pyroelectric lithium tetraborate is of space group of *I*4_1_*cd*, and a rather complex tetragonal crystal with 104 atoms per unit cell, with dimensions a = 9.479 Å and c = 10.290 Å [8,9,10,11,12,13]. While several band structure calculations exist [14,15], there is no uniform consistency in the predicted band structure. Recent density functional theory (DFT) band structure calculations [14] suggest that the hole mass is larger than the electron mass. In other words, the dispersion of the valence bands should be small compared to the expected dispersion of the conduction bands. Only recently [5,7] has there has been experimental confirmation of any of the key predictions of the calculated band structure, beyond the band gap [5,7,16,17,18] and some of the optical properties [15].

**Figure 1:**
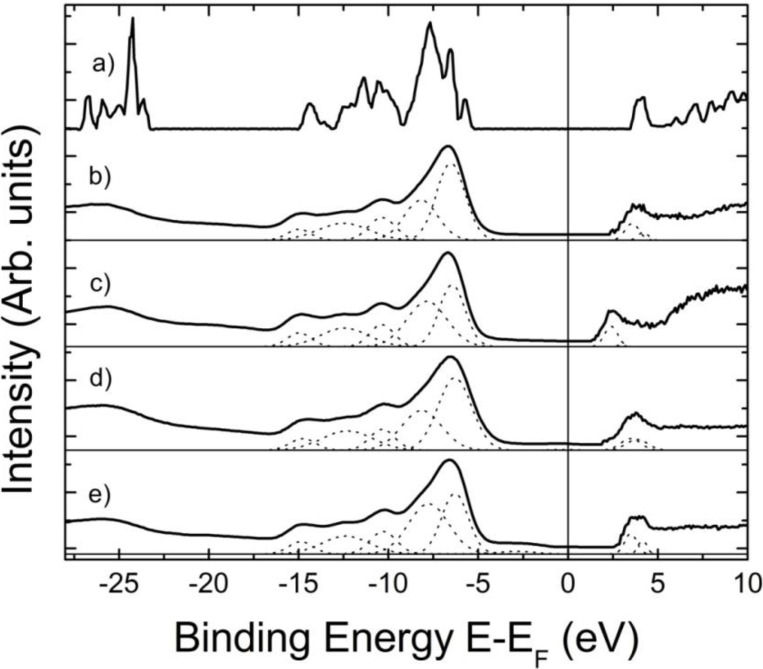
A comparison of the combined experimental photoemission (left) and inverse photoemission (right) data, in E-E_F_, with theoretical expectations. The theoretical density of the bulk band states of crystalline Li_2_B_4_O_7_; (**a**) obtained by the LDA PW1PW is adapted from Islam *et al*. [14]. The combined experimental photoemission (left) and inverse photoemission (right) data for Li_2_B_4_O_7_(100), with the in-plane component of the incident light **E** for photoemission oriented along [011]; (**b**) and [010]; (**c**) are shown along with the data for Li_2_B_4_O_7_(110) with the in-plane component of the incident light **E** for photoemission oriented along [001]; (**d**) and [110]; (**e**) For the photoemission, the photon energy is 56 eV and the synchrotron light is incident at 45 degrees with respect to sample normal. The electrons were either collected along the surface normal (photoemission) or incident along the surface normal (inverse photoemission). The sample temperature for these measurements was 623 ± 5 K, to reduce surface charging. Adapted from [7].

Both Li_2_B_4_O_7_(100) and Li_2_B_4_O_7_(110) exhibit, in combined photoemission and inverse photoemission studies, a density of states that qualitatively agrees with the results from model bulk band structure calculations for Li_2_B_4_O_7_ [10,11], as seen in Figure 1. For Li_2_B_4_O_7_(100) the band gaps obtained from combined photoemission and inverse photoemission are 10.1 ± 0.5 eV and 8.9 ± 0.5 eV with the in plane component of **E** aligned along the [011] and [010] orientations, respectively. For Li_2_B_4_O_7_(110), the band gaps are 9.8 ± 0.5 eV in both the [001] and [110] orientations. In general, the combined photoemission and inverse photoemission measure the direct band gap, but as final state spectroscopies. Consequently, perfect agreement with a ground state calculation, such as density functional theory, is usually not possible and unlikely beyond the qualitative; although the agreement as seen here between experiment and the prior band structure calculations [14] is generally quite good.

The Fermi level is placed slightly closer to the conduction band edge in the combined experimental photoemission and inverse photoemission spectra, as seen in Figure 1. This indicates that both the Li_2_B_4_O_7_(100) and Li_2_B_4_O_7_(110) surfaces are n-type, although (100) is more n-type than (110). While we have not measured the majority carrier, the Fermi level placement is consistent with the known bulk properties where the majority of defects seen in these Li_2_B_4_O_7_(100) and Li_2_B_4_O_7_(110) were oxygen vacancies [12,19]. Point defects comprising of isolated oxygen vacancies, and to a smaller extent isolated lithium vacancies, with a very small trace of Cu impurities were evident in electron paramagnetic resonance (EPR) and electron-nuclear double resonance (ENDOR), in agreement with prior measurements [12]. The existence of these oxygen vacancies is also consistent with a placement of the Fermi level closer to the conduction band edge as a result of oxygen vacancies.

The general prediction that the hole mass should be far greater than the electron mass, based on the calculated band structure [14], is supported by the experimental band mappings of the Li_2_B_4_O_7_(100) and Li_2_B_4_O_7_(110) surfaces [7]. So far there has been no evidence of dispersion of the occupied bands; that is to say there is no strong indication of wave vector dependent changes in binding energy, with either photon energy or emission angle, for the filled states [7]. The absence of any such dispersion suggests that the occupied states for both the Li_2_B_4_O_7_(110) and Li_2_B_4_O_7_(100) surfaces are characterized by a very heavy mass. From the absence of dispersion in the photoemission data [7], we can assign a lower bound to the hole effective mass of 10 [m*/m_e_]. This lower bound is limited by the feature widths in photoemission, and the limited wave vector and energy resolution, and of course the effective mass could be much greater.

The absence of dispersion with photon energy, in angle-resolved photoemission, is often attributed to the conservation of two dimensionality of state, that is to say a surface state. We do not believe that this possible attribution of the states seen in photoemission to surface states is a reasonable assignment in the case for the Li_2_B_4_O_7_(100) and Li_2_B_4_O_7_(110) surfaces. These occupied states, aside from the surface state identified below, do not fall into a gap of the projected band structure and indeed are heavy mass states that simply do not disperse very much. Nor can the problem with the dispersion (the absence of observed dispersion) be related to disorder at the surface: the inverse photoemission exhibits band dispersion of the empty states consistent with the surface Brillouin zone [7].

**Figure 2:**
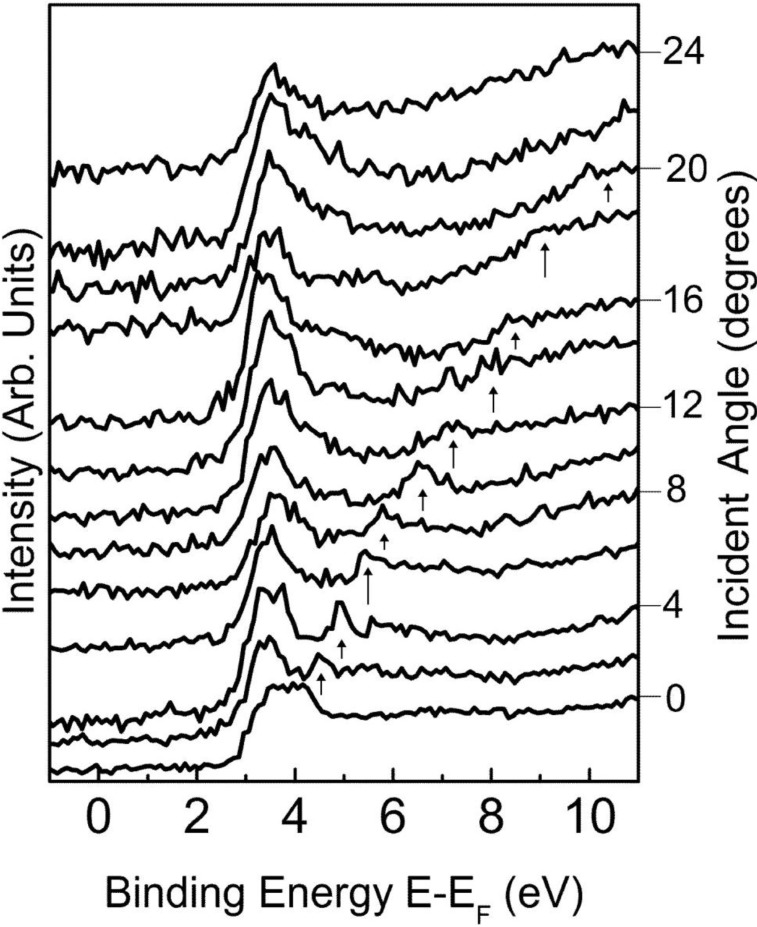
The incidence angle dependent inverse photoemission spectra for the Li_2_B_4_O_7_(110) with increasing incidence angle (wave vector) along the [110] direction. The image state wave vector dependent (incidence angle) dispersion (see text) is indicated by the arrows. The unoccupied state binding energies are given in terms of E-E_F_. Adapted from [7,17].

In contrast to the results garnered from the angle-resolved photoemission, dispersion is evident in empty states observed in the angle-resolved inverse photoemission results [7], obtained as a function of electron incidence angle (*θ*), as seen in Figure 2. The parallel momentum (*k*_||_) in inverse photoemission can be derived as follows from the electron kinetic energy and the incidence angle (*θ*) with respect to the surface normal [20,21]:

(4) $$k_{\left|\right|}=\sqrt{\frac{2m}{h^{2}}E_{Kin}}\text{sin}(\theta )=0.51198\sqrt{E_{Kin}\{eV\}}\text{sin}(\theta )\{{Å}^{-1}\}$$

**Figure 3:**
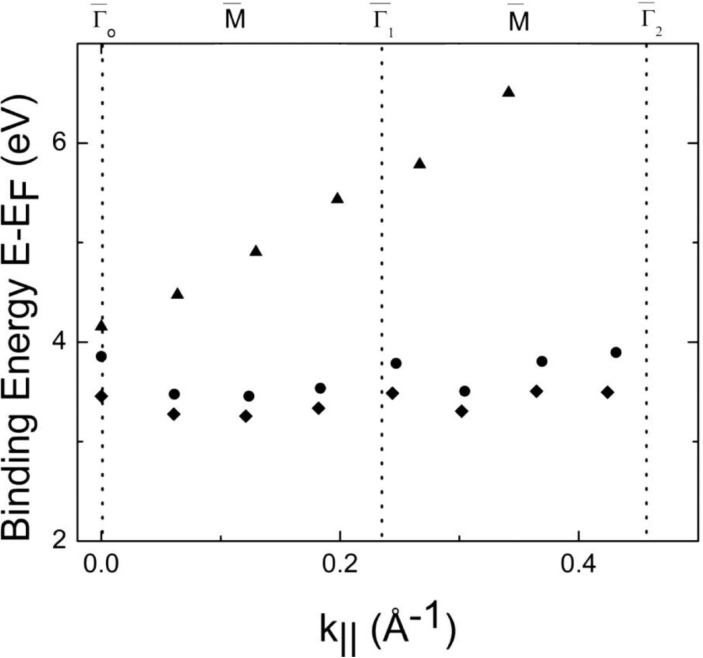
The unoccupied state binding energies versus the surface parallel wave vector are mapped for the Li_2_B_4_O_7_(110) along the [110] direction. (♦) denotes the lowest unoccupied molecular orbital, (●) a higher energy empty state, and (▲) an image state. The surface Brillouin zone critical points are denoted at top. Adapted from [7].

Despite the much lower resolution of inverse photoemission, one is able to observe dispersion of the bands at the conduction band minimum, as shown in Figure 2 and plotted in Figure 3. The periodicity in the dispersion is consistent with the expected Brillouin zone of the Li_2_B_4_O_7_(110) surface, for the states near the conduction band minimum, as plotted in Figure 3, along both the [001] and [110] directions. Again, this could be related to the surface electronic structure, particularly as inverse photoemission is notoriously surface sensitive, but these states also do not fall into a gap of the projected band structure.

**Figure 4:**
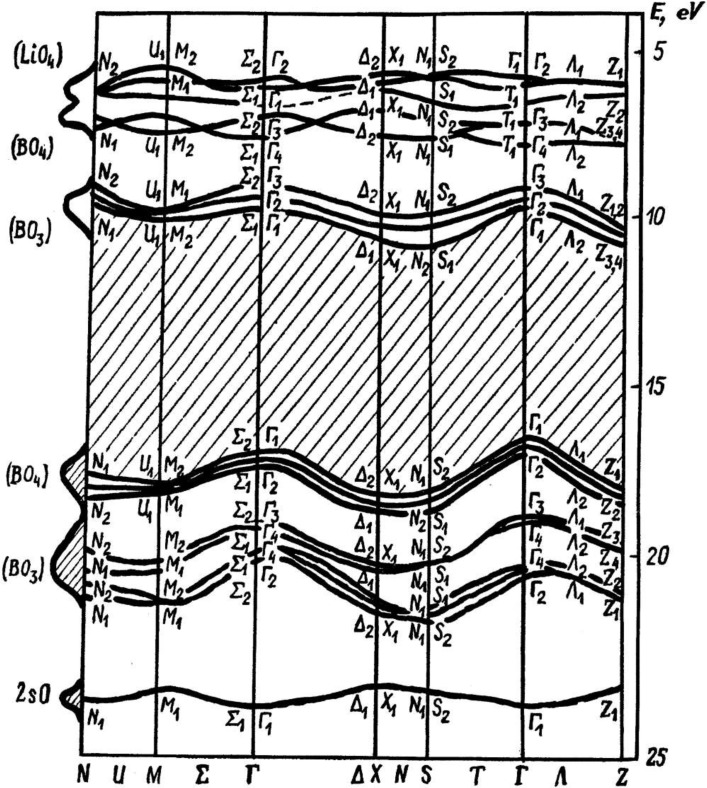
The Calculated electronic bulk band structure of lithium tetraborate Li_2_B_4_O_7_. Adapted from [15].

The trend of the dispersion of the states at the conduction band minimum, towards the Fermi level with increasing wave vector away from the center of the surface Brillouin zone, is qualitatively the opposite trend of one of the DFT calculation [14]. The semi-empirical LDA calculations of Adamiv and coworkers [15] provided a much smaller band gap than observed in the combined photoemission and inverse photoemission (Figure 1). The trend of the dispersion in the semi-empirical LDA calculations of Adamiv and coworkers [15] (Figure 4), however, towards the Fermi level with increasing wave vector away from the center of the surface Brillouin zone is the same qualitative behavior seen with the unoccupied states near the conduction band minimum in the band mapping obtained from inverse photoemission [7], as illustrated in Figure 3.

The qualitative agreement in the unoccupied band dispersion relationship was obtained between experiment and a band structure of Li_2_B_4_O_7_ calculated on the basis of crystallographic analysis of phonon parameters, and structural fragments of BO_3_, BO_4_, LiO_4_ and LiO_6_. [15,16]. The 2s-, 2p_x_-, 2p_y_-, 2p_z_- B and 2s-Li orbitals served as a basis set for the secular equations. The 2s-O orbitals were introduced within a first order perturbation approach [15]. Screening effects were taken into account to obtain better agreement between calculated energy gaps and experimental data. An additional correction (caused by deeper energy bands) was introduced using optical functions obtained from the fundamental reflection spectra [15]. The energy band structure (including a third coordination sphere) was calculated with a final correction according to the optical functions, to better achieve a full quantitative agreement. This energy band structure is shown in Figure 4.

In this latter calculation of the band structure [15], a maximal correction (up to 1.5 eV) was necessary for electron bands originating from the LiO_6_ octahedral. There is a significant dispersion of the band structure is observed for the valence zone top along Γ–X–S direction that is determined by the BO_4_ clusters but this is not observed in the experimental band mapping. A valence band originating from BO_3_-orbitals is situated below the latter. The 2s-O states are responsible for a nearly dispersionless band lying 3 eV below the bands of BO_3_ in origin. The states at the conduction band minimum are built of the antibonding BO_3_-orbitals. Antibonding BO_4_-orbitals and LiO_4_-orbitals are involved in formation of the next grouping of bands (with energies higher than previous ones by about 4 eV). The LiO_6_ clusters produce a high energy band which is not generally considered to be involved in the optical transitions.

The BO_3_ and BO_4_ structural fragments in the Li_2_B_4_O_7_ single crystals exhibit mainly covalent bonding, while the LiO_4_ and LiO_6_ fragments are basically ionic. The covalent bond is determined by the 2p-B and 2p-O strongly hybridized orbitals with a small asymmetry towards boron and oxygen atoms. The 2p-O orbitals serve as bonding orbitals between different clusters of the Li_2_B_4_O_7_ system as well. The hybridization is substantially diminished in the middle of the LiO_4_ and LiO_6_ structural components and, therefore, the polarizability of the chemical bonds increases because of a separation of electron charge between the 2s-Li and 2p-O orbitals. Generally the electron densities calculated in this manner are in very good agreement with the combined photoemission and inverse photoemission, as noted above.

From inverse photoemission, the electron effective masses for the unoccupied Li_2_B_4_O_7_(110) and Li_2_B_4_O_7_(100) states near the conduction band minimum has been estimated [7] to be in the region of −0.15 ± 0.1 [m*/m_e_].

A nearly parabolic lighter mass band is also seen to disperse independent of the surface Brillouin zone, as shown in Figure 2 and Figure 3, again along both the [001] and [110] directions of the surface Brillouin zone for the Li_2_B_4_O_7_(110) surface. This lighter mass band observed at binding energies (E-E_F_) well above the Fermi level for the Li_2_B_4_O_7_(110) surface is an image state, as discussed later. In fact, there was no image potential state observed for the Li_2_B_4_O_7_(100) surface.

### 2.1. Surface states within the gap of the projected bulk band structure

**Figure 5:**
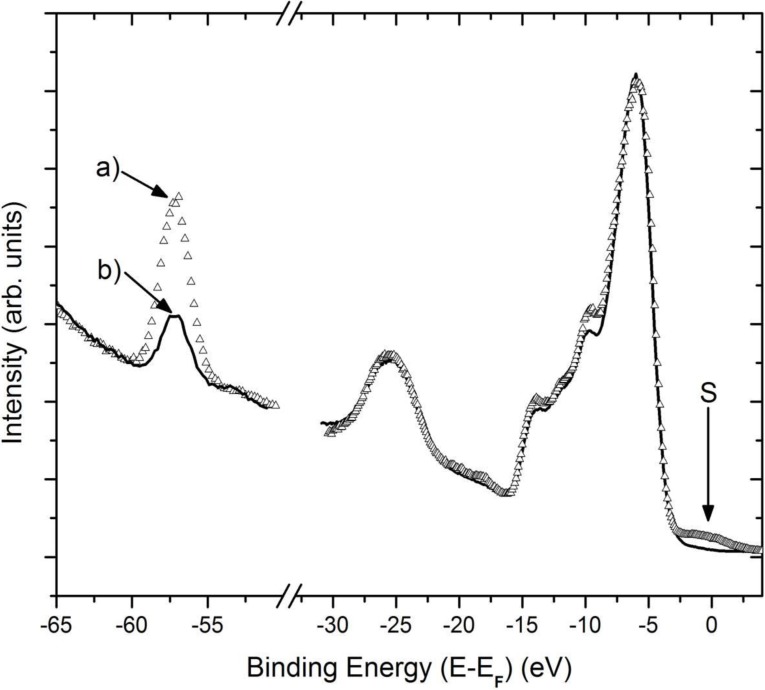
Experimental light polarization dependent photoemission spectra for Li_2_B_4_O_7_(110) oriented along [001] with regards to the in-plane component of **E**. The photon energy is 95 eV with the photoelectrons collected along the surface normal; the light incidence angle was (**a**) 70 degrees {∆ ∆ ∆} or (**b**) 45 degrees {^______^} with respect to surface normal. The arrow indicates the position of the surface contribution (S) to the occupied density of states above the bulk density of states valence band maximum. The sample temperature for these measurements was 623 ± 5 K, to reduce surface charging. Adapted from [7,17].

While the valence band maximum and band gap is in reasonable agreement with expectations, from angle resolved photoemission, there is evidence for states within the valence band maximum to conduction band minimum gap (the insulator band gap), as seen in Figure 5. In general, there is little light polarization dependence observed in the photoemission spectra taken for both the Li_2_B_4_O_7_(100) and Li_2_B_4_O_7_(110) surfaces [7]. As seen in Figure 5, the light polarization dependent photoemission in the valence band region of the Li_2_B_4_O_7_(110) surface exhibits few differences between a light incidence angle of 70°, placing the electric vector **E** more along the surface normal and a light incidence angle of 45°. Yet with a light incidence angle of 70°, there is a small density of states within the gap placed close to the Fermi level, as seen in Figure 5. In this region of energy near the Fermi level, within the bulk band gap and below E_F_, the presence of surface states has been marked by an “S” in Figure 5.

As these Li_2_B_4_O_7_(110) surface states fall into the gap of the projected bulk band structure (Figure 1a) [14], we can initially conclude that these observed occupied states are, in fact, true surface states [7]. The observed intensities of these surface states in photoemission are clearly affected by light polarization (Figure 5) and these surface states are likely of s or p_z_ character (with z along the surface normal), given that they are enhanced with incident light where the electric vector **E** is more along the surface normal. Certainly for the Li_2_B_4_O_7_(110) surface, the possibility of surface states must be given serious consideration as a Li 1s surface to bulk core level shift has been observed for this surface [17].

### 2.2. The Li 1s surface-to-bulk core level shift

**Figure 6:**
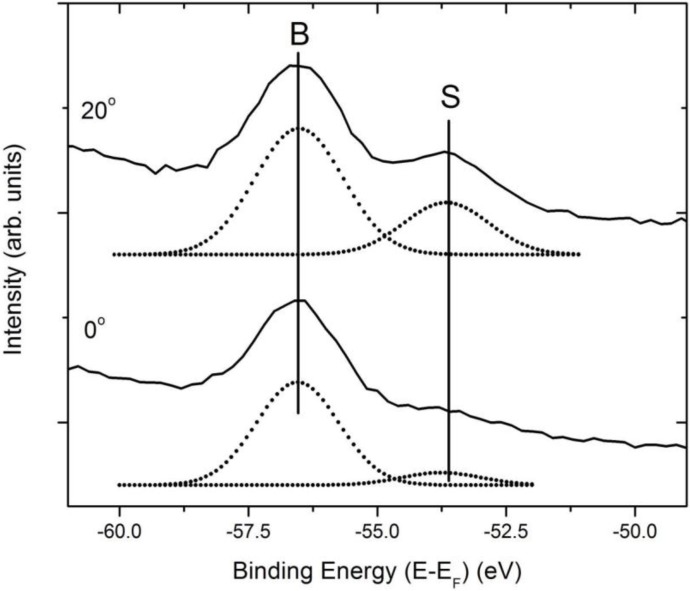
Angle-resolved photoemission spectra of Li_2_B_4_O_7_(110) in the region of the Li 1s core level, for photoelectron emission angles of 20° and 0°. In both cases, the in-plane component of **E** was oriented along [001] with a light incidence angle 45° and with photon energy of 95 eV. The figure shows spectra with the instrumental background and secondary electron tail subtracted from the spectra with fittings to the Li 1s bulk and surface peaks, denoted B (bulk component contribution) and S (surface component contribution), respectively. The sample temperature for these measurements was 623 ± 5 K, to reduce surface charging. Adapted from [17].

Through the use of angle-resolved photoemission spectra taken from Li_2_B_4_O_7_(110) with the in-plane component of **E** oriented along [001] and at a photon energy of 95 eV, we have identified the Li 1s core level photoemission feature, as shown in Figure 5 and Figure 6. After corrections for photovoltaic charging (as discussed later (*vide infra*) and elsewhere [5,7,16,17,18]), we place the Li 1s shallow core level binding energies at −56.7 ± 0.4 to −56.5 ± 0.4 eV depending on the surface termination (crystal orientation). These values are largely dominated by the bulk Li 1s core level binding energy contributions because of the normal photoelectron emission geometry. By increasing the electron emission angle, two components can be easily resolved for the Li_2_B_4_O_7_(110) surface at −56.5 ± 0.4 eV and −53.7 ± 0.5 eV, as illustrated in Figure 6.

From prior studies of the surface-to-bulk core level shift [22,23], the reasonable expectation is to observe an increase in the intensity of the surface component of the core level with increasing emission angle. The intensity of the Li 1s core level component for the Li_2_B_4_O_7_(110) surface at the smaller binding energy of −53.7 ± 0.5 eV is shown to increase with increasing emission angle and thus we assign the Li 1s component at this binding energy as the surface component. The feature at a higher binding energy of −56.5 ± 0.4 eV is attributed to the Li 1s bulk component for Li_2_B_4_O_7_(110).

The temperature dependence of this surface-to-bulk core level shift we later exploit to study possible surface piezo-electric effects (*vide infra*).

### 2.3. The image state at the Li_2_B_4_O_7_(110) surface

Image states are characteristic of clean, flat, largely defect free surfaces. The absence of any image states for the Li_2_B_4_O_7_(100) surface is consistent with the presence of defects at this surface; defects which are very likely surface oxygen vacancies, as has been noted above. The presence of an image state for the Li_2_B_4_O_7_(110) surface (Figure 2 and Figure 3), indicates that this surface is largely defect free with a very flat surface potential. We note that the Li_2_B_4_O_7_(110) surface image state dispersion is not periodic in nature and, in fact, is seen to be simply parabolic along both the [001] and [110] directions of the surface Brillouin zone. For this to occur, the surface potential of the Li_2_B_4_O_7_(110) surface must be so flat that the image state is not perturbed by the surface crystallography, and disperses almost independently of the surface Brillouin zone (Figure 3).

While, as we noted above, the Li_2_B_4_O_7_(110) surface is distinguished by a surface state within a gap of the projected bulk band structure (Figure 5), this surface also exhibits very little light polarization dependence of the bulk valence band states [7,17]. It is this same surface with a small but significant off-axis pyroelectric effect [5], as discussed below. Both effects may combine to reduce the surface potential variations leading to a parabolic image potential state. What is also evident is that the image state for the Li_2_B_4_O_7_(110) surface possesses a very light effective mass of m*/m_e_ = 0.06 ± 0.02. Such a light mass image state is much less likely to be perturbed by the surface potential, as is observed [7].

## 3. Piezoelectricity and Off-Axis Pyroelectricity in Lithium Tetraborate

It has been known for some time that single crystals of Li_2_B_4_O_7_ exhibit piezoelectric properties [24,25] as well as pyroelectric properties [25,26]. The piezoelectric properties may make lithium tetraborate attractive for surface acoustic wave (SAW) device applications [24,27,28,29,30,31]. Defining

P_l_ = d_lij_σ_ij_ (1b)

E_l_ = −g_ljk_σ_jk_ (1c)

P_l_ = −e_ljk_ε_jk_ (2b)

E_l_ = −h_lij_ε_ij_ (2c)

where again P_l_ is the electric polarization vector, σ_ij_ is the mechanical stress, ε_jk_ is the elastic deformation, d_lij_ are the piezoelectric coefficients (10^−12^ C/N), e_ljk_ are the piezoelectric stress constants (C/m^2^), g_ljk_ are the piezoelectric constants (m^2^/C), while h_lij_ are the piezoelectric deformation constants (10^9^ N/C). The piezoelectric coefficients have now been pretty well established and there is convergent agreement, as summarized in Table 1.

Table 1 indicates that the piezoelectric constants of Li_2_B_4_O_7_ are placed between piezoelectric constants of LiNbO_3_ (d_15_ = 74 × 10^−12^ C/N and d_33_ = 18.9 × 10^−12^ C/N) and piezo-quartz (d_11_ = 2.3 × 10^−12^ C/N). The piezoelectric measurements [25,34] show high values for the hydrostatic piezoelectric constants g_33_ = (0.200 − 0.223) Vm/N and g_h_ = (0.100 − 0.179) Vm/N providing some indication of the interest in Li_2_B_4_O_7_ as a possible pressure sensor. The piezoelectric constant g_33_ of Li_2_B_4_O_7_ is among the largest known [25]. The piezoelectric constants d_31_ and d_32_, in the temperature range from 4 K to 900 K, decrease monotonically with decreasing temperature [36].

**Figure 7:**
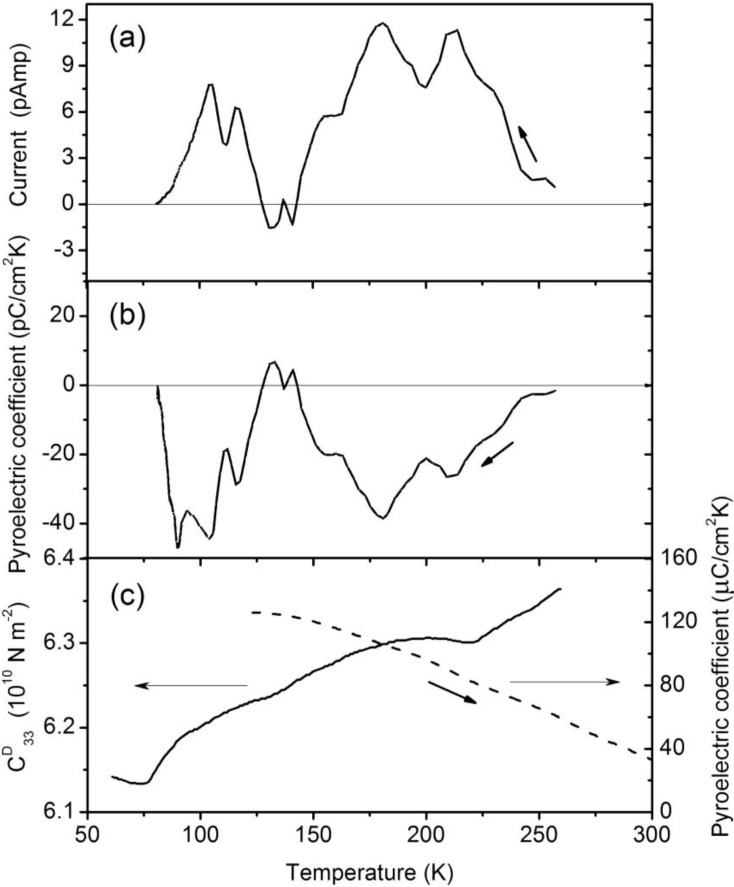
(**a**) Pyroelectric current in the cooling cycle for the Li_2_B_4_O_7_ single crystal in the <110> direction, at a cooling rate of roughly 0.25 degrees/sec, adapted from [5]; (**b**) temperature dependence of the pyroelectric coefficient in the cooling cycle for the Li_2_B_4_O_7_ single crystal in the [110] direction, also adapted from [5]; (**c**) temperature dependence of the elastic stiffness constant $C_{33}^{D}$ for the Li_2_B_4_O_7_ single crystal along the polar *c*-axis (solid line), adapted from [37] and the polar [001] pyroelectric coefficient (dashed line), adopted from [25].

Although perhaps better known as a piezoelectric, lithium tetraborate, as indicated at the outset, does have an appreciable pyroelectric coefficient along the [001] direction, in the region of 100 K to 250 K [25,26], as plotted in Figure 7c. The [110] and [100] crystal directions are orthogonal to the polar [001] direction for the tetragonal crystal lattice and thus candidates for the study of an off-axis pyroelectric effect, which makes lithium tetraborate a nice playground for the demonstration of a secondary pyroelectric effect.

**Table 1:** A summary of the experimentally determined piezoelectric characteristics of Li_2_B_4_O_7_ single crystals.

Constants	Units	[32]	[31]	[33]	[28]	[34]	[25]	[35]
d_15_	(10^-12^ C/N)			3.2		6.33	-	8.07
d_33_				19.8		20.4	24.0	19.4
d_31_				−3.2		−2.02	−4.8	−2.58
e_15_	(C/m^2^)	0.39	0.36	0.36	0.472	0.35		
e_31_		0.24	0.19	0.19	0.290	0.38		
e_33_		0.93	0.89	0.87	0.928	0.88		
g_15_	(m^2^/C)	0.082						
g_31_		−0.032						
g_33_		0.231						
h_15_	(10^9^ N/C)	4.95						
h_31_		3.34						
h_33_		12.9						

### 3.1. The off-axis pyroelectric effect observed for lithium tetraborate

There is a pyroelectric current along the [110] direction of stoichiometric Li_2_B_4_O_7_ so that the pyroelectric coefficient is nonzero [5]. From the current and rate of change in temperature, the approximate pyroelectric coefficient along the [110] direction (Equation 3) has been determined [5]. Along the polar [001] direction, the pyroelectric coefficient *p*_i_ is about 125 μC/m^2^·K at 120 K [25]. Along the orthogonal [110] direction, the pyroelectric coefficient *p*_i_ reaches a maximal value of about 0.2 to 0.4 μC/m^2^·K [5], as illustrated in Figure 7, and is clearly much smaller than along the <001> direction of spontaneous polarization. Indeed, the pyroelectric coefficient along the <110> direction is some 300 to 1000 times smaller than the conventional pyroelectric coefficient measured along the polar <001> lithium borate crystallographic direction in the temperature region of 70 K to 250 K. The pyroelectric coefficient *p*_i_ values along the [110] direction remain qualitatively similar in their temperature dependence as obtained from the currents measured from a range of heating and cooling rates.

As in some prior measurements [26], strong variations with temperature in the pyroelectric current and associated pyroelectric coefficient along the [110] direction have been measured [5], as shown in Figure 7. Indeed, the pyroelectric coefficient does not show the same temperature dependence along the [110] direction as has been described [25,26] along the [001] direction, as indicated in Figure 7. The fact that the measured pyroelectric currents and resulting pyroelectric coefficients along the [110] direction differ qualitatively from those measured along the [001] polar direction [25] is compelling evidence that the measured pyroelectric coefficient along the [110] direction is not the result of a crystal miscut and therefore cannot be a projection of the expected [001] pyroelectric current off the polar axis [5].

The pyroelectric current provides dramatic relative changes in the pyroelectric coefficient along the [110] direction with temperature. There are large decreases in magnitude of the off-axis pyroelectric coefficient at about 80 K, 130 K and 240 K (Figure 7b). These temperatures are close to the observed anomalies (Figure 7c) in the elastic stiffness observed at 75, 125 and 215 K [37]. While the elastic constant ${\text{C}}_{33}^{\text{D}}$ decreases with decreasing temperature, reaching a minimum at about 75 K, these anomalies in the elasticity have been observed not only along the polar <001> direction, but also along other crystallographic directions, although significantly smaller magnitude [37]. This qualitative agreement between elastic constant anomalies and the magnitude of the off-axis pyroelectric coefficient suggests that the nonzero pyroelectric coefficient observed along the [110] direction is a result of anharmonic dipole oscillations or asymmetric dipole canting. An off-axis pyroelectric effect would not be expected to be as significant when the lattice is particularly soft, as may occur in the temperature regions near the elastic stiffness anomalies observed at 75, 125 and 215 K [37]. This is expected for a secondary pyroelectric effect, where temperature dependent crystal lattice deformations are permitted to occur [5].

**Figure 8:**
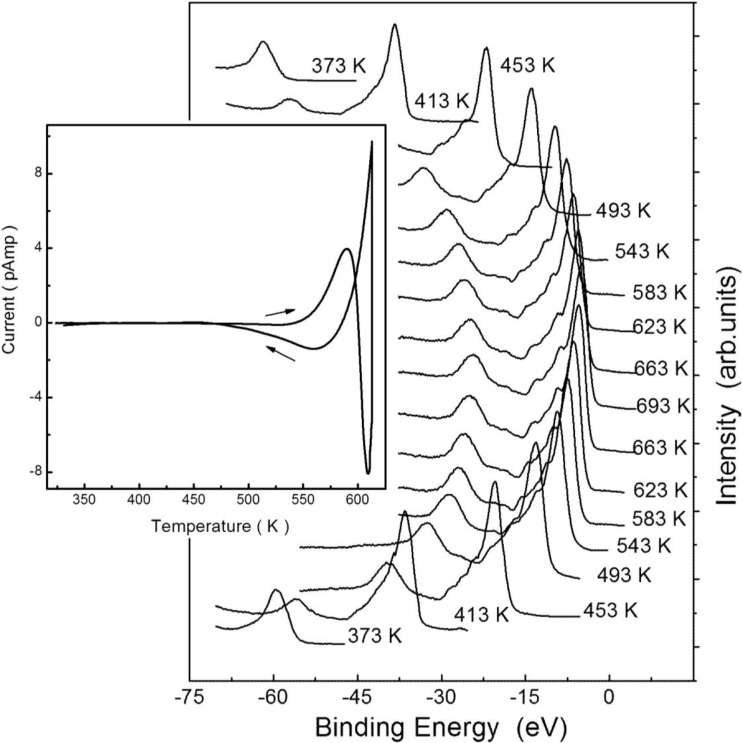
The photoemission spectra from Li_2_B_4_O_7_(110) surface for a succession of temperatures in a heating-cooling cycle (from bottom to top). The photoemission spectra were taken at a photon energy of 56 eV with electrons collected along the surface normal. The measured currents, due to trapped charges, in the [110] direction of lithium tetraborate single crystal with increasing and decreasing temperature (as indicated) in the region of 300 to 600 K are shown in the inset. Adopted from [5].

**Figure 9:**
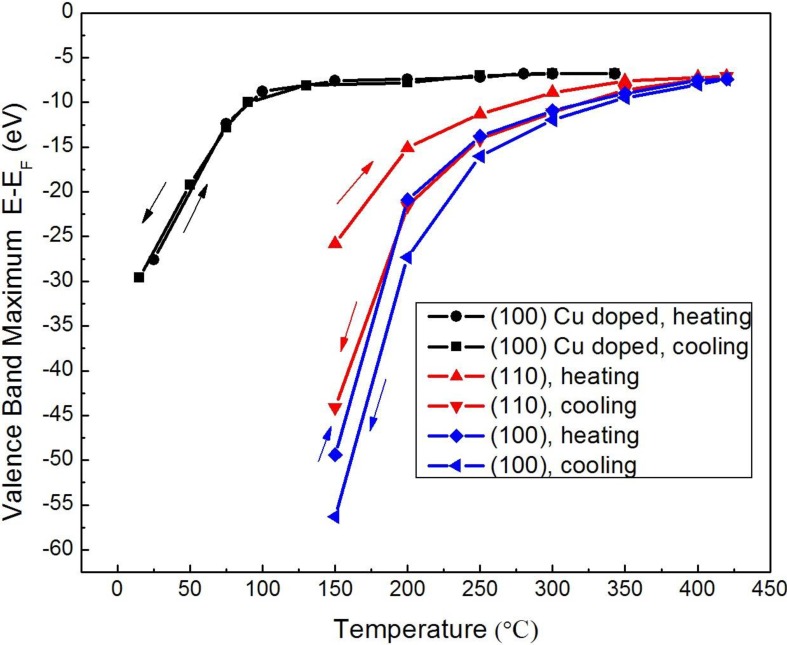
Photovoltaic charging of Li_2_B_4_O_7_(110) (red), Li_2_B_4_O_7_(100) (blue) and Cu doped Li_2_B_4_O_7_(100) or Li_1.998_Cu_0.002_B_4_O_7_(100) (black) as measured from the position of the valence band maximum. Spectra were taken, as in Figure 8, with both heating and cooling and the shifts in the valence band maximum recorded in terms of the apparent binding energy with respect to the Femi level E-E_F_.

Confirmation of an off-axis pyroelectric effect for much higher temperatures is also evident in the photovoltaic charging. A surface charge or surface electric field might not be possible to measure directly using traditional transport measurements but can be observed in photoemission by exploiting the surface photovoltage effect [5,38,39,40,41,42]. The photovoltage charging, shown in Figure 8 and plotted in Figure 9, regrettably cannot be directly compared with the pyroelectric measurements (Figure 7), as illumination is required and such surface photovoltage measurements would tend to work best for a lithium borate surface that is largely defect free [43]. Measurements of the surface charging were essential in any case [5,7,17,18], as the electronic structure measurements reported in the prior section require suppression of surface charging to be a valid measure of electronic structure [7]. We note, in passing, that this is the reason why most of the electronic structure measurements reported in section 2 were done at 623 K, where surface photovoltage charging was found to be negligible [18] and the Li_2_B_4_O_7_(110) and Li_2_B_4_O_7_(100) surface exhibited a density of states that qualitatively resembles that expected from the model bulk band structure of Li_2_B_4_O_7_ [5,7,17,18], as seen in Figure 1.

Below 500 K, the surface photo-voltaic charging is both temperature and time dependent, particularly at the (110) surface. There is clearly hysteresis in the photovoltaic charging observed in photoemission. Although the photovoltaic charging is greater for the (100) surface than the (110) surface, the hysteresis is larger for the (110) surface, as determined from the apparent position of the valence band maximum plotted in Figure 9. This hysteresis in the surface photovoltaic charging, as measured by the valence band maximum, is consistent with the observation of a pyroelectric current along the (110) direction at the much lower temperatures of 70 to 250 K (Figure 7). Not all such indications of surface charge accumulation can be considered consistent with pyroelectricity, however.

In the region of 600 K, there is a huge increase in the absolute magnitude of the current with increasing and decreasing temperature, as shown in Figure 8. The currents generated in the region of 600 K are likely the result of trapped charges or charge trapping point defects [5], with the onset of decreasing or increasing conductivity, respectively, as indicated in the inset to Figure 8, and may have little to do with the more conventional pyroelectric effect. Indeed, the data suggests trapped charges are released with increasing temperature and charge trapping occurs with decreasing temperature [5,19]. The fact that there are two temperature dependent current regimes, in the region of 500 to 600 K, indicates trapped charges may be associated with both lithium and oxygen point defects [19]. The charge trapping or trapped charge release may be the result of ionized defect sites of opposite charge for lithium and oxygen defects, respectively, leading to the generation of currents of opposite sign. This would be expected for oxygen and lithium vacancy defects with different charge trap potentials [5].

### 3.2. Evidence for possible surface pyroelectric/piezoelectric effects

**Figure 10:**
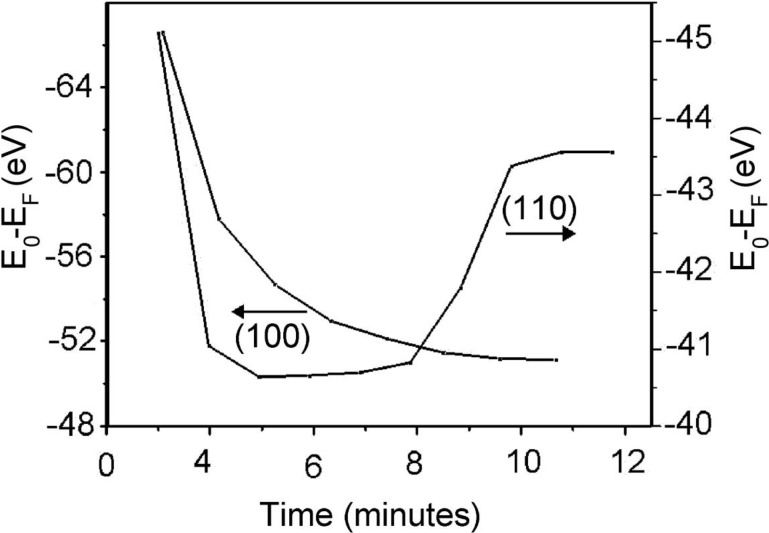
The change of the magnitude of the apparent O 2 s binding energy, with time, indicative of the surface photovoltaic charging of the Li_2_B_4_O_7_(110) surface following a temperature increase to 390 K compared to the Li_2_B_4_O_7_(100) surface following a temperature increase to 420 K. Adapted from [5].

The hysteresis in the photovoltaic charging is not only temperature dependent, but time dependent. The measured effective binding energy (E-E_F_), for the oxygen 2 s shallow core at a binding energy of −26.0 ± 0.6 eV at 623 ± 5 K (Figure 1), increases (in terms of the absolute value) with decreasing temperature, below 600 K. At temperatures below 500 K, using the oxygen 2 s shallow core as a benchmark this observed change in binding energy (and associated photovoltaic charging) can be understood as establishment of a steady state surface temperature and surface conductivity. At the (110) surface, there is not only an increase (in terms of the absolute value) in the effective binding energy, but this is followed by a decrease (in terms of the absolute value) in the effective binding energy later in time, as plotted in Figure 10. This latter absolute value decrease in the effective binding energy later in time is observed at the (110) but not the (100) surface (Figure 10) and this time dependent hysteresis effect is increasingly more evident at lower temperatures. As this occurs in a region where there is little or no bulk current, with changes in temperature (Figure 8), this suggests that, while the surface photovoltage effect is initially dominated by the establishment of a steady state surface temperature and surface conductivity, the surface charge density D_i_ is later altered by the local electric field resulting from the surface photovoltage effect. In other words, at the (110) surface, there are time dependent changes in surface charge density D_i_.

**Figure 11:**
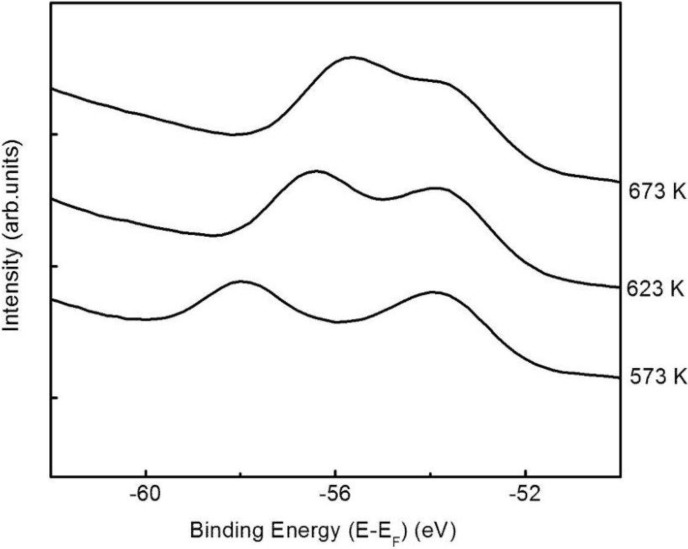
The change in the surface-to-core bulk core level shift with decreasing temperature indicative of differences in the surface photovoltaic charging at the surface and bulk for the Li_2_B_4_O_7_(110) surface.

The valence band features and shallow oxygen 2 s core level shift rigidly together, in apparent binding energies, characteristic of uniform surface photo-voltaic charging in photoemission. This is not the case for the lithium shallow core level. The lithium shallow 1s bulk core level component shifts in binding energy in “lock-step” with the valence band features, with changing temperature, but in the region of 570 K to 670 K, the surface component of the lithium 1s core level, by comparison, shows little evidence of surface photovoltaic charging as indicated in Figure 11. This difference in charging between the surface and bulk components of the Li 1s core level suggests greater conductivity at (110) surface or in the vicinity of the surface lithium sites for temperatures in the region where defect charge trapping occurs, *i.e*., 623 ± 5 K. This greater surface conductivity is supported by the luminescence data discussed later. Because of the differences in apparent surface charging at the (110) surface or in the vicinity of the surface lithium sites, a high temperature surface pyroelectric effect in this temperature regime, while unlikely, cannot *a priori* be excluded. Since the surface is more conductive than the bulk, as suggested by Figure 11, a surface pyroelectric effect must be considered extremely unlikely in the temperature region of 500 to 670 K.

The time dependent hysteresis (Figure 10) is likely a result of a pyroelectric effect leading to a changing surface charge density and charge trapping, as well as possibly an additional surface piezoelectric effect. Certainly, a surface piezoelectric effect can occur at some (but likely not all) surfaces perpendicular to the direction of spontaneous polarization for lithium tetraborate. This too adds credence to the idea [2,5] that the pyroelectric coefficients *p*_i_ that include the secondary pyroelectric effect likely have some tensor character and would probably be more accurately expressed as a third order tensor *p*_ijk_.

## 4. Metal Doping of Lithium Tetraborate

The undoped Li_2_B_4_O_7_ resistivities are on the order of 10^10^ Ω·cm [44], but doping could both suppress pyroelectricity and increase transport, ideally electron transport as the hole mass is quite large, as indicated by the band structure [7]. In comparing the photovoltaic charging of the (100) surface termination for Cu doped and undoped Li_2_B_4_O_7_ (Figure 9), it is clear that doping suppresses not only the surface photovoltaic charging but also hysteresis. While the surface charging at the (100) surface of Li_2_B_4_O_7_ is significantly greater than observed at (110) surface, the Cu doping plays a role in reducing the surface photovoltage effects. With Cu doping of Li_2_B_4_O_7_, while the surface photovoltaic charging is much diminished, the density of states observed with combined photoemission and inverse photoemission remains similar to that observed for the undoped material, except in the vicinity of the conduction band edge [45].

The first issues associated with the doping of the Li_2_B_4_O_7_ single crystals to consider are those connected with a valence of impurity and site location in the crystal lattice. In the case of doping by Cu and Ag it is also probably very important to take into account the large differences between the impurity ionic radii, *i.e.*, r_Cu+_ = 0.96 Å and r_Ag+_ = 1.13 Å, and the host site radii, r_Li+_ = 0.68 Å and r_B3+_ = 0.16 Å. There is no guarantee that a dopant impurity ion Аg^+^ will occupy a B^3+^ ion site and much to suggest it will not, because of the large difference in ionic radii, as well as differences in valence.

Generally, electron paramagnetic resonance (EPR) and electron-nuclear double resonance (ENDOR) studies in Li_2_B_4_O_7_ have focused on Cu^+^ [15,19,46,47], Co^2+^ and Mn^2+^ [48,49], impurities substituting for lithium and vacancy-related defects produced by neutron irradiation at room temperature. The Cu impurity centers in the Li_2_B_4_O_7_ lattice generally adopt the univalent Cu^+^ ion state [46,47,50,51,52,53,54], independent of the valence of the initial chemical copper source additive agent used for Cu doping.

Copper impurity ions substitute for lithium ions in the Li_2_B_4_O_7_ lattice [19,46,47,50,51,52,53,54], with most of them being in the monovalent charge state prior to the X-ray irradiation. Heating above room temperature restores the pre-irradiation distribution of copper charge states. Indeed, thermoluminescence data indicates that the recombination process Cu^2+^ + e to Cu^1+^ + hν occurs in the region of 362 K or less [46]. These ground state Cu^+^ (3d^10^) impurity ions convert to Cu^2+^ (3d^9^) ions during the irradiation as they trap “free” holes from the valence band [19] or (alternatively) the electron of the Cu^2+^ exciton resides in the nearest appropriate lattice defect site [46]. The Cu impurities are extremely robust and stable in the 1+ state, even in the nominally Li_1.998_Cu_0.002_B_4_O_7_, both as-grown [19,46,47,50,51,52,53,54] as well as after irradiation at room temperature with X-rays [50].

It is possible that the Cu atoms occupying the Li sites will act as donors [45] and a more heterogeneous distribution of donor sites would account for the more gradual increase in the conduction band edge density of state away from the Fermi level seen in the inverse photoemission [45]. If either the surface or bulk donor state density increases with Cu doping then the surface photovoltaic charging should diminish compared to the undoped Li_2_B_4_O_7_(100) surfaces, as is observed.

If the Cu impurities actually increase the number of hole traps, then, in spite of the heavy hole mass expected from the band structure [7], the resulting decrease in hole carrier mobilities should decrease the conductivity. There is also the possibility that the Cu^1+^ impurity ion traps an electron to go to a transient Cu^0^ state, as suggested by the thermoluminescence [46] and cathodoluminescence [55,56] of Li_2_B_4_O_7_:Cu. If either process occurs with appreciable probability at the surface as well, then the surface photovoltaic charging should increase compared to the undoped Li_2_B_4_O_7_(100) surfaces.

Can these impurity states, introduced by dopants, be observed more directly?

### 4.1. Optical properties

**Figure 12:**
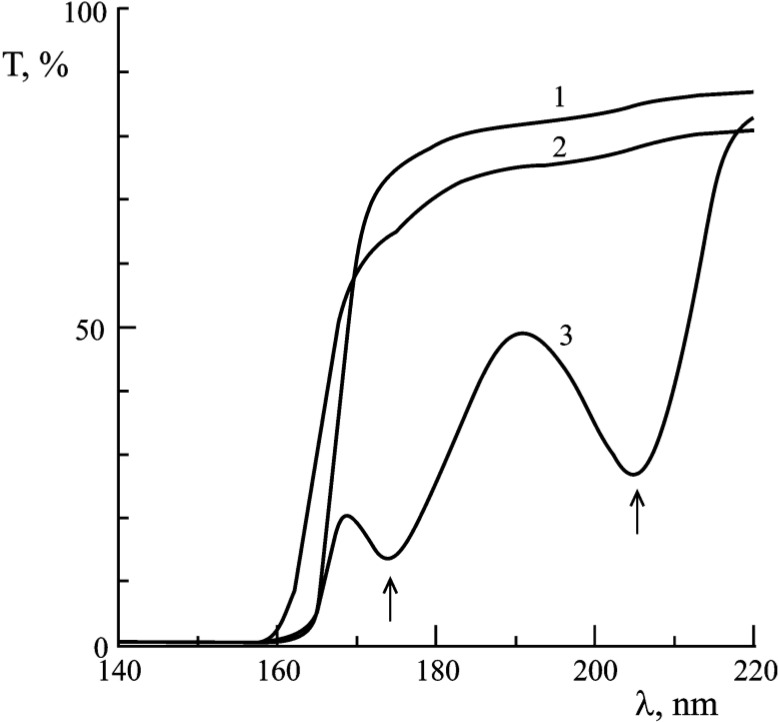
The transmission spectra of Li_2_B_4_O_7_ single crystals at a temperature of 290 K: 1—undoped; 2—Cu doped; 3—Ag doped. Adapted from [50].

Li_2_B_4_O_7_ single crystals are transparent in the range of 165–6000 nm and the fundamental absorption maximum is placed at about 133 nm [57]. The transmission spectra [50] of the undoped as well as Cu and Ag doped Li_2_B_4_O_7_ single crystals are shown in Figure 12. The values of the direct optical band gap energy of undoped Li_2_B_4_O_7_ obtained by extrapolation of the absorption coefficient to zero absorption in the k^2^ = f(hν) plot was E_g_ (opt) = 7.4 eV [15,50], somewhat less than the band gap of 9.8 ± 0.5 eV (with a range from 8.9 ± 0.5 eV to 10.1 ± 0.5 eV) obtained from the combined photoemission and inverse photoemission [7], as discussed previously.

For the undoped and Cu and Ag doped Li_2_B_4_O_7_ single crystals, the transmission spectra do not include any absorption bands in the range of 900–300 nm [50], at a temperature of 290 K. For Li_2_B_4_O_7_:Cu, the transmission spectrum (Figure 12, curve 2) exhibits a broad low-intensity band in 300–167 nm region at a temperature of 290 K [52]. With Ag impurities in Li_2_B_4_O_7_, two intensive absorption bands can be observed near the characteristic absorption edge of the Li_2_B_4_O_7_, namely at 205 and 174 nm (Figure 12, curve 3) [50].

For the case of Li_2_B_4_O_7_:Cu single crystals, the absorption bands of Cu^+^ ions lying near 5 eV correspond to the 3d^10^ → 3d^9^4s electron transitions. For Li_2_B_4_O_7_:Ag single crystals, the corresponding adsorption transition for the Ag^+^ ions is 4d^10^ → 4d^9^5 s. But variations can occur due to the splitting of these levels caused by the crystal field. In free Cu^+^ and Ag^+^ ions, these transitions are generally considered symmetry or parity forbidden, but they can take place in many crystals, like Li_2_B_4_O_7_, due to symmetry breaking as a result of odd symmetry (transverse optical) vibrations of the crystal lattice. The absorption bands due to the Cu^+^ ions in Li_2_B_4_O_7_ exist in sites surrounded by deformed octahedral oxygen. Octahedral oxygen environment is consistent with the evidence, discussed above, that Cu^+^ ions are located at specific locations within the Li_2_B_4_O_7_ crystal lattice, and not at random interstitial sites. With a substitution of Li^+^ ion sites, the oxygen environment about the Cu^+^ ions must be a deformed tetrahedron [46].

**Figure 13:**
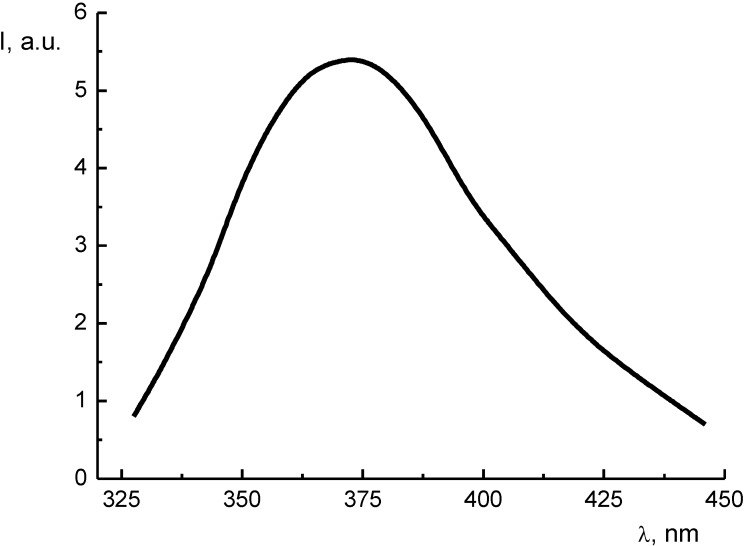
The thermoluminescence spectrum of Li_2_B_4_O_7_:Cu and Li_2_B_4_O_7_:Ag single crystals after X-irradiation, obtained for the 150 K peak, from [50].

On the basis of the results of the thermoluminescence, X-ray luminescence and absorption spectroscopy studies (Figure 13) of Li_2_B_4_O_7_:Cu, and Li_2_B_4_O_7_:Ag single crystals, some of us [50] have proposed a mechanism for the formation of A^0^ (Cu^0^ or Ag^0^) centers of luminescence with the participation of growth defects. The A^0^ centers play a dominant part in the accumulation of the light yield under irradiation. The luminescence occurs by means of the energy transfer to a self-trapped exciton in the Li_2_B_4_O_7_, crystal lattice followed by radiative decay (annihilation) [50]. Thus, the luminescence scheme in Li_2_B_4_O_7_:Cu, Ag single crystals can be represented as follows:

Li_2_B_4_O_7_:A^+^→Irradiated→Li_2_B_4_O_7_:(A^0^+h)→Heat→Li_2_B_4_O_7_:(A^+^+STE)→ →Annihilation→Li_2_B_4_O_7_:A^+^+hν (5)

**Figure 14:**
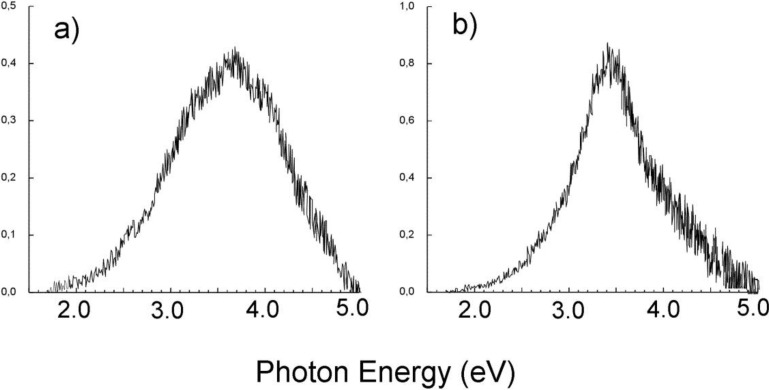
Cathodoluminescence spectra at 295 K of Li_2_B_4_O_7_ single crystals undoped (**a**) and doped with Cu (0.015%) (**b**). Adapted from [55].

It is useful to compare the cathodoluminescence spectra of Li_2_B_4_O_7_:Cu with other luminescence spectra such as X-ray luminescence, photoluminescence, radioluminescence, and especially thermoluminescence. As shown in Figure 14, the cathodoluminescence spectrum of undoped Li_2_B_4_O_7_ single crystals, taken at 295 K, has a maximum at 3.65 eV (340 nm), but contains contributions from a luminescence band at 2.2 eV (564 nm). These cathodoluminescence spectra are very similar to the radioluminescence [58], X-ray luminescence spectra at 293 K [57] and to some extent similar with the thermoluminescence spectrum [46].

We find the cathodoluminescence spectrum of Li_2_B_4_O_7_:Cu single crystals (0.015 at.% Cu) does, however, differ slightly from the luminescence spectra obtained by other excitation mechanisms. For example, the cathodoluminescence spectrum maximum, at 295 K (Figure 14b) is at 3.50 eV (355 nm). For other types of luminescence, the excitations tend to occur in a region near 3.35 eV (370 nm) [46,50,59,60,61] and the shape of the luminescence spectra can differ drastically. In the case of Li_2_B_4_O_7_:Cu, the superposition of some luminescence bands in the cathodoluminescence spectra can be clearly seen: components with maxima at 3.40 eV (365 nm) and 3.55 eV (350 nm) (Figure 14b). Overall, analysis of all the cathodoluminescence spectra provides compelling evidence that the luminescence band of Li_2_B_4_O_7_ is connected with self-trapped excitons near crystal defects [57,62,63].

### 4.2. Carrier lifetimes

The carrier lifetimes or the decay times τ are important for the understanding of the role of dopants like Cu in the suppression of the surface voltaic charging. The cathodoluminescence line widths, which include a strong surface component, suggest that electron lifetime is, in fact, much longer for the Li_2_B_4_O_7_:Cu single crystals than the undoped Li_2_B_4_O_7_ single crystals [55]. This may be taken as strong evidence of the very real possibility of an increase in surface and, possibly, bulk conductivity with copper doping. The broad band photoluminescence was observed for undoped Li_2_B_4_O_7_ crystals, with photo-excitation above 7.5 eV, and the decay luminescence consists of two components with time constants τ < 1 ns and τ ~ 8.5 ns, as well as a slow component in the microsecond time range [64]. The emission at 3.35 eV arising from the Cu^+^ ion 3d^9^4s → 3d^10^ transitions in Li_2_B_4_O_7_:Cu single crystals, with 5 and 7 eV photoexcitation, has a decay time of 25 μs, at 297 K [65]. Our estimations of the τ parameter for the slow component decay time of the cathodoluminescence [55] is τ > 300 ns for undoped crystals and τ > 50 μs for doped Li_2_B_4_O_7_: Cu crystals.

This difference between the decay time of 25 μs seen with photoluminescence and a decay time τ > 50 μs observed in cathodoluminescence for Li_2_B_4_O_7_:Cu crystals means that secondary charge carriers, both electrons and holes (*e* and *h*), are free to move for some time at the surface of Li_2_B_4_O_7_:Cu single crystals. If we accept the mean free lifetime of the secondary charge carriers is τ = 50 μs and the effective electron mass is m*/m_e_ = 0.15 [18], then the mobility may be estimated to be μ = eτ/m* = 1.8 × 10^14^ cm^3/2^·g^−1/2^. Because of the very light mass image state m*/m_e_ = 0.06 [18], the surface mobility may be estimated to be even larger, at about μ = eτ/m* = 4.4×10^14^ cm^3/2^·g^−1/2^.

This enhanced carrier mobility at the surface is consistent with the generally greater surface conductivity evident in the surface photovoltaic charging, as discussed above. The increased surface mobilities and carrier lifetimes, we infer from cathodoluminescence, such as occur with Cu doping, certainly explain the quenching of the hysteresis in the surface photovoltage seen at the surfaces of undoped Li_2_B_4_O_7_(100) and Li_2_B_4_O_7_(110), as evident in Figure 9.

## 5. Experimental Section

In nature, Li_2_B_4_O_7_ occurs as a clear, colorless mineral as inclusions of diomignite in pegmatite [66]. Sastry and Hummel were the first [67] to crystallize lithium tetraborate (Li_2_B_4_O_7_) from a congruent melt. Single crystals of Li_2_B_4_O_7_ were first grown by Garrett and coworkers [68]. The Li_2_B_4_O_7_ single crystals used here were grown from the melt by the Czochralski technique as described elsewhere [15,69]. The [110] and [100] crystals have been cut with a miscut of no more than 0.5°, as determined by X-ray diffraction [5,11]. The pyroelectric measurements along the [110] and [100] directions were performed in a manner similar to prior studies [25,26] over a range of heating rates from 0.015 to 0.4 K/sec, as described elsewhere [5]. At the lowest temperatures (50–70 K), the heating and cooling rates deviate from the linear, and these deviations have been taken into account in the analysis. Regardless, variations in the heating rate from experiment to experiment were not found to significantly alter the measured pyroelectric coefficients [5].

The temperature dependent angle-resolved photoemission spectra were obtained using linearly polarized synchrotron light dispersed by a 3m toroidal grating monochromator [7,17,18,70,71], at the Center for Advanced Microstructures and Devices (CAMD) [72]. The measurements were made in an ultra-high vacuum (UHV) chamber employing a hemispherical electron analyzer with an angular acceptance of ± 1°, as described elsewhere [70,71]. The photoemission experiments were undertaken with a light incidence angle of 45° with respect to the surface normal, unless stated otherwise. The photoelectrons were collected along the surface normal throughout. The photoemission was conducted over a range of temperatures from 250 to 700 K, but the binding energies are referenced to the Fermi level established at temperatures greater than 623 K, where surface charging was found to be negligible [5,7,17,18]. The location of the Fermi level was determined via angle-resolved photoemission using tantalum films in electrical contact with the samples [5,7,17,18]. The inverse photoemission was conducted in a separate vacuum system, as described elsewhere [7,20], with a k-resolved band mapping of the unoccupied levels obtained by changing the incidence angle.

The reference of the observed binding energies to the Fermi level for Li_2_B_4_O_7_(110), as done here, differs from the sometimes common practice of assigning binding energies with respect to the valence band maximum for lithium tetraborate [14]. Prior studies of lithium tetraborate also have assigned their binding energies with respect to the chemical potential or Fermi level [73]. We chose the latter convention for this investigation, *i.e.*, citing binding energies in terms of E-E_F_.

In the photoemission experiments, after various combinations of argon ion sputtering and annealing, the surface was found to be ordered, stoichiometric and free of contamination. The surface ordering was confirmed by the presence of a dispersing (E versus k_||_ dependent) band structure in angle-resolved inverse photoemission with the critical points that match the expected surface periodicity. The absence of surface contamination at the Li_2_B_4_O_7_(110) surface prepared for the temperature dependent photoemission studies is evident in the photoemission spectra taken at higher photon energies [5,7,17] and from clear evidence of a light mass image state [7] in the angle-resolved inverse photoemission. Although the unoccupied band structure of Li_2_B_4_O_7_(110) surface, mapped out in inverse photoemission [7], is consistent with the lattice constants of the (110) surface, reconstructions of the surface cannot be excluded. Because of the extremely dielectric nature of these crystals, low energy electron diffraction intensity versus voltage I(V) surface structural analysis was not possible in the temperature range where the pyroelectric currents were most evident (well below room temperature).

While the X-ray diffraction shows that the material is well oriented and single phase, electron paramagnetic resonance (EPR) and electron-nuclear double resonance (ENDOR) revealed three different point defects in the undoped crystals [19]. From greatest amount to least, these different defects were isolated oxygen vacancies, isolated lithium vacancies, and a trace of Cu impurities. These isolated point defects, amounting to between 2 and 5 ppm in total, were not sufficient to degrade the dielectric properties of our crystals, and there was insufficient current to observe any power law conductivity. Indeed very little current was generated with intense neutron irradiation of Li_2_B_4_O_7_ crystals enriched to 95 at % ^6^Li and 97.3 at % ^10^B (isotopes with very high neutron capture cross-section) at more than 500 V applied bias, indicating very little dark current is possible in Li_2_B_4_O_7_ crystals with natural abundance of Li and B, in the absence of irradiation or illumination, as is the case in the crystals studied here.

Cathodoluminescence of the Li_2_B_4_O_7_ and Li_2_B_4_O_7_:Cu samples was performed at room temperature in the pulsed regime (with a pulse duration of 3 μs with repetition rate of 20 Hz). The energy of electron beam was 9.5 keV, the current in the beam was 200 μA, the diameter of electron beam on the sample was 1 mm, and the angle of incidence on the sample surface was 30°. The depth of electron penetration can be calculated according to the empirical formula [74,75]:

(6) $$R=\frac{2.76\times 10^{-2}AU^{5/3}}{\rho Z^{8/9}}\text{cos}30^{\circ }\;(\text{μm})$$

where *Z* it the effective atomic number, *A* is the effective atomic weight appropriate to the effective *Z*, *U* the accelerating voltage, and ρ, density. For Li_2_B_4_O_7_ crystals, where *Z* = 7.3, *A* = 12, ρ = 2.44 g/cm^3^, the penetration depth *R* of electrons under an accelerating voltage of 9.5 kV and at the angle of incidence of 30° does not exceed 0.85 μm.

## 6. Conclusions

We find a pyroelectric current and hysteresis in the surface photovoltaic charging along the [110] direction of stoichiometric Li_2_B_4_O_7_. The pyroelectric coefficient is roughly 10^3^ smaller than along the [001] direction of spontaneous polarization but nonzero. Abrupt decreases in the pyroelectric coefficient along the [110] direction can be correlated with anomalies in the elastic stiffness ${\text{C}}_{33}^{\text{D}}$ contributing to the concept that the pyroelectric coefficient is not simply a vector but has qualities of a tensor, as expected. The concept of a secondary pyroelectric effect, considered as a very real possibility for decades [2], has now been conclusively demonstrated in at least one pyroelectric material.

The time dependent surface photovoltaic charging suggests that pyroelectric and piezoelectric effects that occur at the (110) surface may be more significant than those that occur at the (100) surface. These effects are complicated by the fact that the surface conductivity may well differ significantly from the bulk due to a light mass image state at the (110) surface, leading to the enhanced surface carrier mobilities at elevated temperatures.

As with all piezoelectric materials, lithium tetraborate is a dielectric, but pyroelectricity is suppressed with Cu doping. It should be noted that because of the very light electron mass, compared to the heavy hole mass, as abstracted from the experimental band structure, it is clear that excitations leading to electron-hole pair creation are unlikely to lead to complete charge collection on a short time scale. This will also apply to current generated by electron-hole pair creation generated by ^6^Li or ^10^B nuclei neutron capture (with large cross-sections for thermal neutrons), although currents have been measured as a result of neutron irradiation for some lithium borates [44,76]. While the asymmetry between electron and hole carrier mobilities is tied to the band structure, carrier lifetimes are seen to increase by a factor of more than 150 with doping, and thus doping could lead to improved charge collection with excitation spectroscopies.
